# Molecular Detection of *Tannerella forsythia* in the Synovial Fluid of a Patient with Knee Osteoarthritis: A Case Report Suggesting Oral–Joint Translocation

**DOI:** 10.3390/diagnostics16101565

**Published:** 2026-05-21

**Authors:** Ivana Milić, Mihovil Plečko, Domagoj Vražić, Domagoj Delimar, Ivan Puhar

**Affiliations:** 1 Dental Polyclinic Zagreb, 10000 Zagreb, Croatia; 2 Department for Orthopaedic Surgery, University Hospital Center Zagreb, 10000 Zagreb, Croatia; 3 Department of Periodontology, School of Dental Medicine, University of Zagreb, 10000 Zagreb, Croatia; 4 Department for Orthopaedic Surgery, School of Medicine, University of Zagreb, 10000 Zagreb, Croatia

**Keywords:** *Tannerella forsythia*, osteoarthritis, periodontitis, synovial fluid, case report

## Abstract

**Background and Clinical Significance:** *Tannerella forsythia* is a Gram-negative, anaerobic bacterium primarily associated with severe periodontal disease. Extraoral infections are extremely rare, and the presence of *Tannerella forsythia* in synovial fluid has not been reported previously. **Case Presentation:** We report a case of a 60-year-old female with grade IV primary osteoarthritis referred for dental evaluation. Clinical and radiographic examination revealed stage IV generalized periodontitis. Subgingival plaque samples were collected after non-surgical periodontal therapy and analyzed using a semi-quantitative real-time PCR targeting the 16S rRNA gene. During planned knee surgery, synovial fluid was obtained and processed using the same molecular protocol. PCR analysis of samples from the deepest periodontal pockets and synovial fluid confirmed the presence of *Tannerella forsythia*, demonstrating its potential dissemination from oral to joint tissues. The postoperative course was uneventful, with no signs of joint infection. **Conclusions**: To the best of our knowledge, this is the first reported detection of *Tannerella forsythia* in the synovial fluid of a patient with osteoarthritis, supporting the possibility of oral–joint microbial translocation. This finding should be interpreted with caution, as the detection of bacterial DNA does not indicate viability or causative involvement. More research is necessary to clarify the mechanisms underlying this association.

## 1. Introduction

Osteoarthritis (OA) is the most common musculoskeletal disorder among middle-aged and elderly populations [1]. It is primarily associated with the destruction of articular cartilage; however, the pathological process affects all joint tissues [2]. As early as 2019, the global number of people with osteoarthritis exceeded 500 million, making OA one of the leading causes of chronic pain and long-term disability in older adults. The incidence and global burden of osteoarthritis continue to increase, driven primarily by population aging and the rising prevalence of obesity [3]. Historically, OA was considered a degenerative joint disease caused by excessive wear and mechanical stress. It is now recognized that this disease is associated with increased production of inflammatory mediators in affected joints, indicating that inflammation plays a key role in osteoarthritis pathogenesis [4].

Emerging evidence suggests that oral pathogens can modulate inflammatory processes in the joints. Several studies have identified bacteria such as *Porphyromonas gingivalis* and *Fusobacterium nucleatum* in the synovial fluid of patients with various forms of arthritis, including rheumatoid arthritis and osteoarthritis, highlighting a potential link between the oral microbiome and joint disease pathogenesis [5,6].

*Tannerella forsythia* is a Gram-negative anaerobic bacterium commonly found in the oral cavity. Along with *Porphyromonas gingivalis* and *Treponema denticola*, it forms the so-called “red complex” present in dental plaque [7]. It is a major periodontal pathogen involved in the development of periodontitis, yet accumulating evidence indicates that it can also cause extraoral infections. Although its role in oral infections is well documented, the presence of Tannerella forsythia in synovial fluid has not been reported previously. *Tannerella forsythia* is fastidious and difficult to cultivate; therefore, molecular methods such as PCR are essential for its detection in clinical samples. This case report highlights the potential systemic impact of periodontal pathogens beyond the oral cavity, including their possible role in osteoarthritis pathogenesis.

## 2. Case Presentation

### 2.1. Patient Information

A 60-year-old female patient diagnosed with grade IV primary OA of the knee was referred from the Orthopedic Clinic of the Clinical Hospital Center Zagreb to the Dental Polyclinic Zagreb for a dental examination and periodontal assessment as part of a larger research study. The patient had a history of degenerative disease of the lumbosacral spine and an L5-S1 disc herniation, for which she was taking Arcoxia (60 mg) and Lyrica (75/150 mg). She had no history of autoimmune disease, systemic infection, or immunosuppressive therapy.

Prior to any clinical procedures, the patient received written information about the study and provided informed consent in accordance with the ethical principles of the Declaration of Helsinki. This case report was prepared in accordance with the CARE (CAse REport) guidelines.

### 2.2. Clinical Findings

At the initial visit, panoramic radiography revealed the loss of 19 teeth and marked horizontal and vertical alveolar bone loss (Figure 1). A detailed periodontal examination was subsequently performed, and periodontal status was assessed. Standard periodontal indices were measured at six sites per tooth. Mean probing depth (PD) was 4.44 mm, mean gingival recession (RE) was 0.67 mm, and mean clinical attachment level (CAL) was 5.11 mm. Oral hygiene status was poor, with a full-mouth plaque score (FMPS, O’Leary) of 83.3% and a full-mouth bleeding score (FMBS) of 93.3%. Based on these clinical and radiographic findings, stage IV generalized periodontitis was diagnosed.

### 2.3. Diagnostic Assessment

On the same day, supragingival plaque and calculus removal were performed. One day after the non-surgical phase of treatment, PCR testing was performed to detect the most common periodontal pathogens (Table 1). Samples were pooled from the deepest periodontal pockets using sterile paper points and sent to the molecular laboratory at Polyclinic Analiza for microbiological analysis. Bacterial detection was performed using a semi-quantitative real-time PCR approach. Results were interpreted according to the validated laboratory protocol and expressed using a semi-quantitative scale (+ to +++), reflecting the estimated bacterial concentration in the sample.

Periodontal samples were vortexed with 500 µL of saline for 10 s to dislodge the bacteria from the paper points. Genomic DNA was subsequently isolated using the NucleoSpin^®^-Microbial kit (Macherey-Nagel, Düren, Germany), as per the manufacturer’s protocol. For real-time PCR, 5.0 µL of the extracted DNA was combined with 25 µL of Power SYBR Green PCR Master Mix (Thermo Fisher Inc., Waltham, MA, USA), 18 µL of sterile water, and 2 µL (20 µM) of a bacteria-specific primer pair in a final volume of 50 µL. Previously published primers targeting the 16S ribosomal RNA gene were tested under optimal conditions and amplification efficiency. The primer concentrations were the same for all assays.

Amplifications were performed on an ABI Prism 7500 Real-Time PCR System (Applied Biosystems, Waltham, MA, USA) using the following cycling parameters: initial denaturation at 95 °C for 10 min, followed by 40 cycles of denaturation at 95 °C for 5 s and annealing at 60 °C for 34 s each. Fluorescence from SYBR-Green binding to dsDNA was measured at each cycle. All amplifications and detections were carried out in a MicroAmp optical 96-well reaction plate. Following amplification, a melting curve analysis (60 °C to 95 °C) was conducted to verify reaction specificity.

### 2.4. Therapeutic Intervention

Following sampling, subgingival scaling and root planing were performed on the same day. The patient received detailed oral hygiene instructions, and a follow-up appointment was scheduled after six months to monitor clinical parameters and evaluate periodontal therapeutic outcomes. Eighteen days after periodontal sampling, the patient underwent total knee arthroplasty (Alloarthroplastica genus) using the EPT Permedica Prime Flex transfer model (F-9, T-E, polyethylene insert D-E 12 mm) (Figure 2A,B).

A sample of synovial fluid was collected during the procedure and processed using validated real-time PCR protocols previously applied for subgingival samples, enabling direct comparison (Table 2). Synovial fluid was aspirated intraoperatively under strictly sterile conditions prior to prosthesis placement. The surgical field was disinfected according to institutional protocol, and the sample was immediately transferred to sterile containers to prevent environmental contamination.

### 2.5. Follow-Up and Outcomes

PCR analysis of samples from the deepest periodontal pockets and synovial fluid confirmed the presence of *Tannerella forsythia* DNA, supporting the possibility of dissemination from oral to joint tissues. Following therapy, partial periodontal improvement was observed, primarily reflected in reduced periodontal inflammation. The patient also reported subjective improvement in joint-related symptoms. At follow-up, no clinical signs of periodontal or joint complications were observed, indicating a stable clinical outcome (Table 3) (Figure 3A,B).

## 3. Discussion

To the best of our knowledge, this case report represents the first documented detection of *Tannerella forsythia* in the synovial fluid of a patient with OA, supporting the possibility of oral–joint microbial translocation. This finding potentially opens a new area of research on the possible link between the oral microbiome and OA pathogenesis, a relationship previously investigated primarily in rheumatoid arthritis and other inflammatory arthritides. However, this should be interpreted as hypothesis-generating rather than evidence of causality, as the presence of bacterial DNA does not confirm microbial viability or a pathogenic role in joint disease.

Previous studies have identified identical bacterial DNA (*Fusobacterium nucleatum*) in the periodontal tissue and synovial fluid of OA patients [6]. Additionally, *Enterococcus faecalis* has been isolated from knee joint tissues in OA patients undergoing surgical procedures, as reported in the literature [8]. Comparable associations between periodontal pathogens and synovial inflammation have also been described in inflammatory arthritides, particularly rheumatoid arthritis, further supporting the concept of oral–joint microbial interactions [9,10]. These observations suggest the possibility of hematogenous dissemination of oral pathogens from periodontal tissues to the synovial space. Similar to previous studies reporting oral bacterial DNA in synovial samples, this case supports the hypothesis of microbial translocation from the oral cavity to the joint environment. However, in contrast to these reports, this study identifies *Tannerella forsythia* in synovial fluid, expanding the spectrum of oral microorganisms potentially associated with OA-related inflammatory processes. Collectively, these findings suggest that oral bacterial DNA in synovial environments may represent a broader phenomenon associated with inflammatory joint conditions, supporting the hypothesis that periodontal pathogens could contribute to systemic inflammatory processes beyond the oral cavity, although the biological significance of this association remains uncertain.

*Tannerella forsythia* was first isolated as a slow-growing, fusiform bacterium in 1979 at the Forsyth Institute from dental plaque samples of patients with periodontitis. It possesses a unique ability to produce the toxic and proinflammatory compound methylglyoxal from glucose. Methylglyoxal can trigger an inflammatory response, leading to periodontal tissue degradation and the release of host components that the bacterium then utilizes as nutrients [11]. *Tannerella forsythia* is considered a major periodontal pathogen, with high prevalence in periodontal pockets of patients with periodontitis [12,13]. Reports of its isolation have been limited, likely due to the fastidious nature of this anaerobic bacterium and the complex conditions required for cultivation [14]. Given that conventional diagnostic methods are challenging, *Tannerella forsythia* often remains undetected [15]. Studies have shown that detection rates derived via culture are significantly lower than those found with PCR, highlighting the importance of molecular diagnostics in epidemiological research and the clinical management of periodontal disease [16]. PCR can detect bacterial DNA in periodontal and synovial samples despite the fastidious growth characteristics of *Tannerella forsythia*.

In this case, PCR testing for the most common periodontal pathogens was performed on samples from a periodontal pocket and synovial fluid. The presence of *Tannerella forsythia* in both sites (+++ and +) suggests the potential for the hematogenous dissemination of oral pathogens and their involvement in joint inflammatory processes in patients with periodontitis and OA. In addition to their individual virulence factors, the periodontal pathogens investigated in this study exhibit synergistic interactions within the subgingival biofilm that promote chronic inflammation and tissue destruction. *Porphyromonas gingivalis*, *Tannerella forsythia*, and *Treponema denticola* form the so-called “red complex,” strongly associated with advanced periodontal disease, while *Aggregatibacter actinomycetemcomitans* is also recognized as an important periodontal pathogen associated with aggressive forms of periodontitis and enhanced inflammatory response. *Prevotella intermedia* contributes to biofilm maturation, dysbiosis, and inflammatory activation within periodontal tissues. Importantly, all periodontal pathogens investigated in this study were Gram-negative bacteria. Unlike Gram-positive bacteria, Gram-negative bacteria possess an outer membrane containing lipopolysaccharides (LPSs), potent stimulators of host inflammatory responses, and produce virulence factors such as proteolytic enzymes and leukotoxins [17]. Mounting evidence supports a connection between periodontitis, chronic low-grade inflammation (LGI), and systemic health. The presence of periodontal pathogens elicits a host’s inflammatory response, activating innate immune mechanisms via phagocytosis and acute inflammatory pathways [18]. Persistent infection triggers the adaptive immune response through T and B lymphocytes, leading to the release of proinflammatory molecules, including interferons, IL-17, TNF-α, IL-1, IL-6, and inflammation-associated enzymes such as collagenases and matrix metalloproteinases. Proinflammatory mediators produced in inflamed periodontal tissue can reach distant tissues and organs via the circulation, potentially initiating or exacerbating inflammatory conditions elsewhere in the body [19]. This systemic perspective is further supported by recent studies, where periodontal inflammation was shown to alter oral microbial composition and inflammatory signaling pathways [20]. In parallel, the disruption of intestinal barrier integrity and gut microbial homeostasis associated with periodontal inflammation has also been described; this supports the concept of an oral–gut inflammatory axis involved in systemic inflammatory response [21]. This cumulative inflammatory burden may help explain how periodontal pathogens contribute to systemic inflammatory diseases, including osteoarthritis, without directly causing joint infection.

Although OA was once considered purely degenerative, it is now recognized that an imbalance in pro- and anti-inflammatory cytokines plays a crucial role in its development, leading to cartilage degradation. One study confirmed that IL-6 and TNF-α serum levels are associated with knee cartilage loss, indicating that low-grade inflammation contributes to OA pathogenesis [22].

Relatively few studies have linked *Tannerella forsythia* infection to the development of other systemic diseases. *Tannerella forsythia* and *Porphyromonas gingivalis* can damage host tissues via enzyme and toxin production, contributing to esophageal cancer development [23]. A case of lung abscess has also been reported in a patient with periodontitis in which *Tannerella forsythia* was identified as the dominant microorganism [24]. Moreover, associations between this bacterium and atherosclerotic cardiovascular disease in patients with periodontitis have been documented [25]. Although extraoral findings of *Tannerella forsythia* remain relatively uncommon, the available evidence suggests that this periodontal pathogen may have systemic relevance beyond the oral cavity. In this context, the detection of *Tannerella forsythia* DNA in synovial fluid in the present case may reflect a potential link between chronic periodontal inflammation and inflammatory processes observed in osteoarthritis.

This case further supports the hypothesis of a link between periodontitis-induced chronic low-grade inflammation and OA pathogenesis. It also has important scientific and clinical implications. First, it expands the spectrum of oral pathogens that can be detected in synovial fluid, highlighting the relationship between oral health and OA. Second, it raises new research questions regarding the role of oral microorganisms in modulating inflammatory processes and cartilage degradation. These findings suggest that timely recognition and effective management of periodontitis might provide additional benefits in preventing or slowing OA progression.

This study has several limitations that should be acknowledged. First, the observation is based on a single case, which limits the ability to draw causal inferences regarding the relationship between microbial factors and joint degenerative processes. Second, quantitative PCR data were not available, and bacterial load was assessed only semi-quantitatively. Third, functional studies assessing bacterial activity in the synovial environment were not performed; thus, the presence of bacterial DNA does not confirm the presence of viable microorganisms or their active involvement in inflammation. However, the detection of *Tannerella forsythia* DNA in synovial fluid using a validated molecular protocol, combined with the absence of clinical signs of septic arthritis, suggests low-grade translocation rather than active joint infection. Overall, the present findings should be interpreted within the broader context of the increasingly recognized association between periodontal disease, systemic inflammation, and osteoarthritis, in which periodontal pathogens may contribute to inflammatory processes rather than act as direct causative agents of joint degeneration.

Given these limitations, future studies should include larger patient cohorts and apply quantitative and genomic approaches, such as quantitative PCR (qPCR) and metagenomic sequencing, to better understand the potential role of oral pathogens in osteoarthritis. This will also clarify the mechanisms underlying microbial translocation and its contribution to low-grade systemic inflammation and joint degeneration.

## 4. Conclusions

The present case highlights a possible oral–joint microbial link, although larger studies using quantitative and genomic approaches are needed to better understand its biological significance. However, detection of bacterial DNA does not confirm microbial viability or causative involvement. Further research could clarify the potential role of oral pathogens in OA pathogenesis and open avenues for new microbiome-targeted therapeutic and preventive strategies.

## Figures and Tables

**Figure 1:**
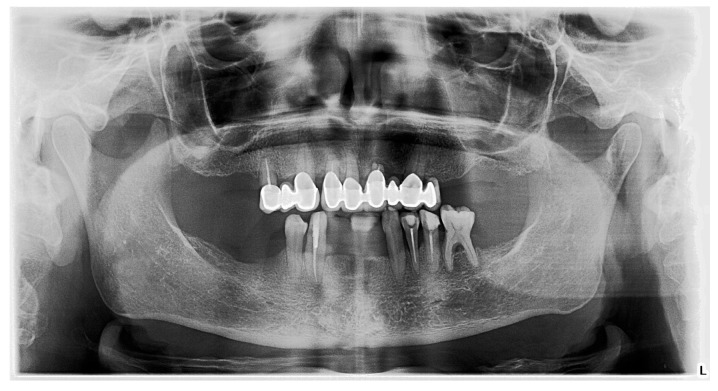
Panoramic radiograph demonstrating a loss of 19 teeth and severe alveolar bone resorption.

**Figure 2:**
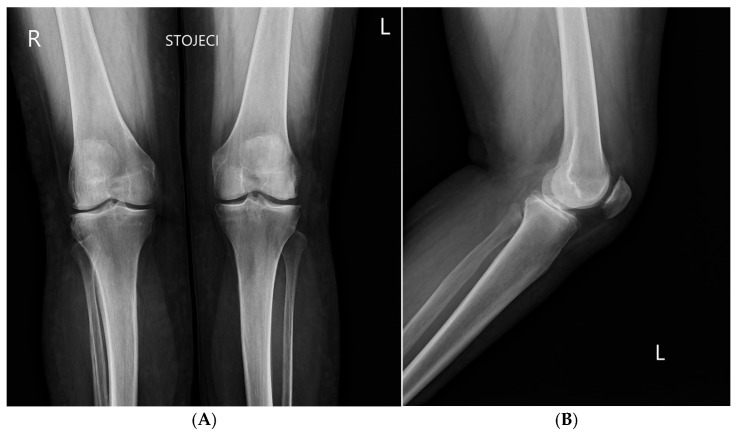
Knee radiograph prior to surgery: (**A**) anteroposterior view and (**B**) lateral view.

**Figure 3:**
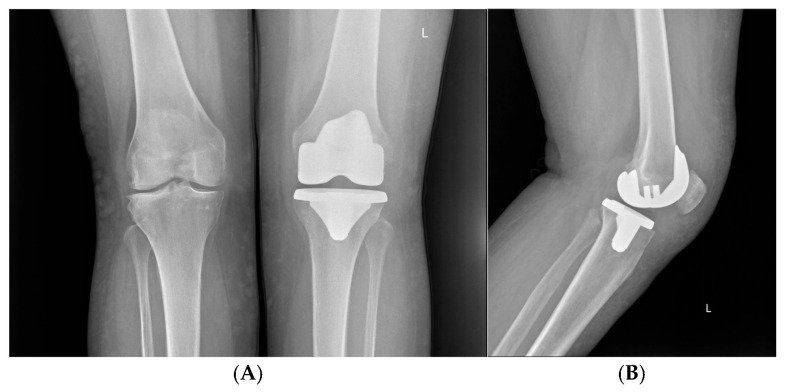
Knee radiograph after the surgery: (**A**) anteroposterior view and (**B**) lateral view.

**Table 1:** PCR for periodontopathogenic bacteria—subgingival sample. Applied Biosystems/RT-PCR method (Thermo Fisher Scientific, Foster City, CA, USA) with SYBR-Green/melting curve analysis (validated according to micro-IDent^®^, Hain Lifescience GmbH (Baden, Germany), CE-IVD and PeriodontScreen Real-TM CE, Sacace Biotechnologies, Como, Italy).

Bacterium	Semi-Quantitative Result	Probable Concentration of Pathogen/mL of Suspended Sample
*Aggregatibacter actinomycetemcomitans*	++	10^4^–10^5^
*Porphyromonas gingivalis*	+	10^3^–10^4^
*Prevotella intermedia*	++	10^4^–10^5^
*Tannerella forsythia*	+++	>10^5^
*Treponema denticola*	++	10^4^–10^5^

**Table 2:** PCR for periodontopathogenic bacteria—synovial fluid sample. Applied Biosystems/RT-PCR method with SYBR-Green/melting curve analysis (validated according to micro-IDent^®^, Hain Lifescience GmbH, CE-IVD and PeriodontScreen Real-TM CE, Sacace Biotechnologies).

Bacterium	Semi-Quantitative Result	Probable Concentration of Pathogen/mL of Suspended Sample
*Aggregatibacter actinomycetemcomitans*	-	<10^3^
*Porphyromonas gingivalis*	-	<10^3^
*Prevotella intermedia*	-	<10^3^
*Tannerella forsythia*	+	10^3^–10^4^
*Treponema denticola*	-	<10^3^

**Table 3:** Timeline of clinical events.

Time Point	Clinical Event
Day 0	Dental and periodontal examination; panoramic radiograph; supragingival plaque and calculus removal.
Day 1	Subgingival sampling for detection of periodontal pathogens (Real-time PCR analysis); subgingival scaling and root planning.
Day 19	Total knee arthroplasty; intraoperative synovial fluid sampling (18 days after periodontal sampling).
Postoperative period	Uneventful recovery; no clinical or laboratory signs of joint infection.

## Data Availability

The raw data supporting the conclusions of this article will be made available by the authors upon request.

## Abbreviations

The following abbreviations are used in this manuscript:

PCR	Polymerase chain reaction
OA	Osteoarthritis
PD	Probing depth
CAL	Clinical attachment level
FMPS	Full-mouth plaque score
FMBS	Full-mouth bleeding score
